# A review of nomenclature in minimally invasive coronary artery bypass grafting—the anarchy of terminology

**DOI:** 10.1093/icvts/ivae204

**Published:** 2024-12-05

**Authors:** De Qing Görtzen, Ferdi Akca

**Affiliations:** Department of Cardiothoracic Surgery, Catharina Hospital, Eindhoven, The Netherlands; Department of Cardiothoracic Surgery, Catharina Hospital, Eindhoven, The Netherlands

**Keywords:** minimally invasive, coronary artery bypass grafting, MIDCAB, LAST, MICS, CABG, TECAB

## Abstract

**OBJECTIVES:**

Since the development of minimally invasive coronary surgery, nomenclature has rapidly grown to distinguish each unique method. The goal of this review was to provide a comprehensive overview of the different terms used for minimally invasive coronary bypass grafting through the years.

**METHODS:**

A literature search was performed in August 2024 using the PubMed electronic database. To extract the best search results: “minimally invasive” and “coronary artery bypass grafting” were used as either keywords or MeSH terms. The term *robotic* was specifically included for a second search. Eligible articles for this review were articles using an abbreviation to describe minimally invasive coronary artery bypass grafting.

**RESULTS:**

A total of 2118 publications on non-robotic minimally invasive coronary procedures and 392 on robotic-assisted techniques were reviewed, describing 40 unique terms for the procedure. Procedures were grouped based on left internal mammary artery harvest and anastomosis methods: mini-thoracotomy for both harvesting and coronary anastomosis (*n* = 586), endoscopic harvest with mini-thoracotomy (*n* = 37), robotic harvest with mini-thoracotomy (*n* = 144) and closed-chest revascularization (*n* = 140). Minimally invasive direct coronary artery bypass grafting was the most studied technique (486 publications, non-robotic and robotic), followed by closed-chest totally endoscopic coronary artery bypass (*n* = 124).

**CONCLUSIONS:**

In conclusion, a wide variety of terms are used within the field of minimally invasive coronary surgery. A total of 40 different terms have been published, each describing certain specifics of the procedure. For anyone involved in the field of minimally invasive surgery, it is important to understand the differences and similarities of these procedures.

## Graphical Abstract

**Figure:**
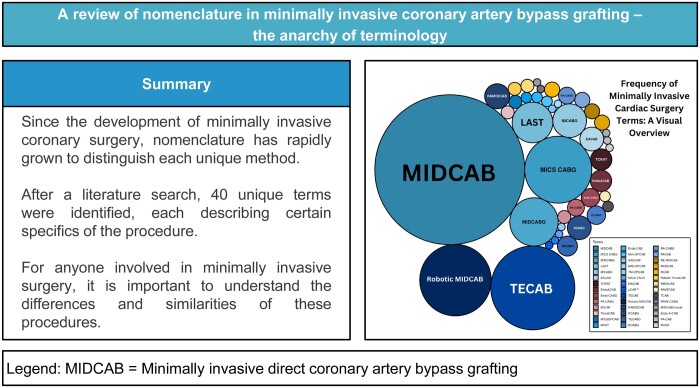

## INTRODUCTION

**Figure 1:**
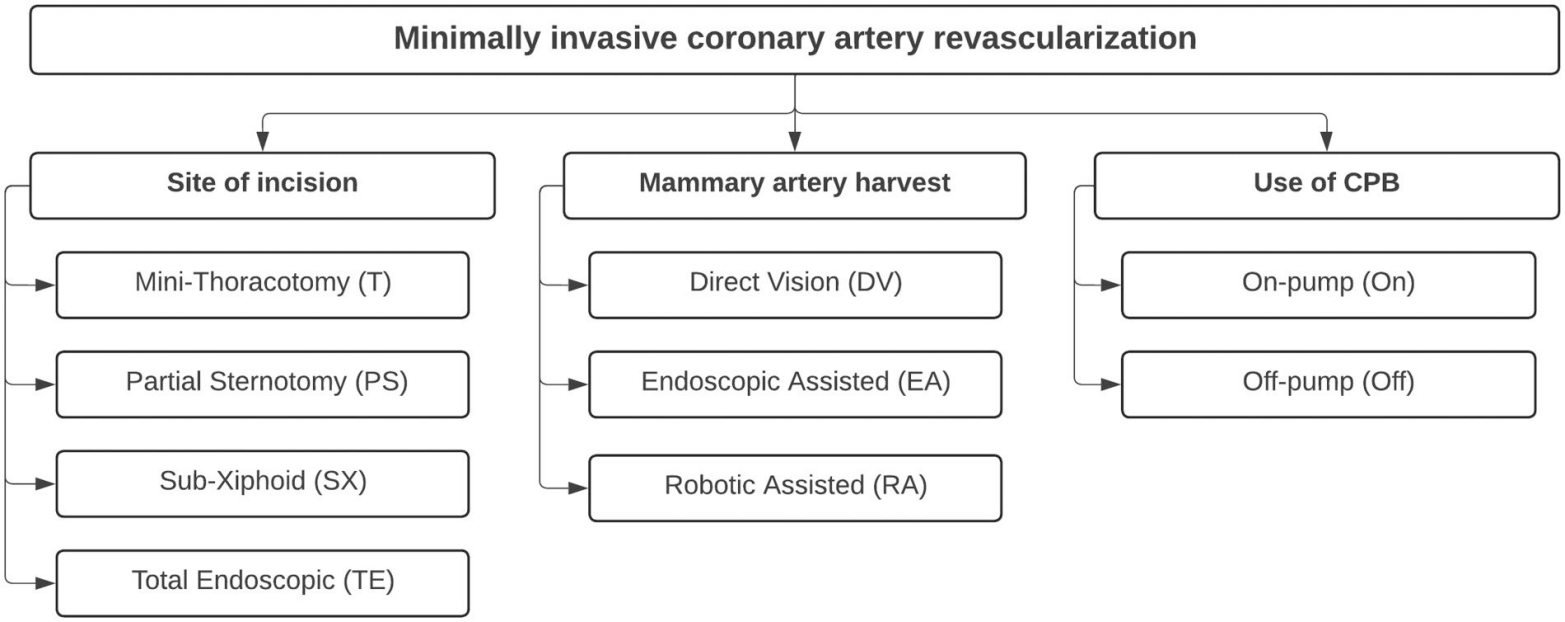
Classification based on site of incision, mammary artery harvesting technique and use of cardiopulmonary bypass. CPB: coronary pulmonary bypass.

Since the beginning of coronary artery bypass grafting (CABG), there has been a continuous development of alternative less invasive approaches, starting as early as 1964 [1–5]. The initial goal was to explore alternate approaches for redo procedures. At first, the sternal-sparing left thoracotomy incision could not be considered minimally invasive. However, it did open the possibility for future less invasive strategies. In the early 1990s, the first mention of the use of the left thoracotomy to perform minimally invasive revascularization was published by Benetti *et al.* [6]. Soon thereafter, the first abbreviation was created in 1996 to distinguish this minimally invasive approach, called the left anterior small thoracotomy (LAST) [7]. It was followed by a general term for minimally invasive direct coronary artery bypass grafting (MIDCAB) [8]. The development of minimally invasive port-access internal mammary artery harvest opened up the possibility of robotically assisted procedures (closed-chest totally endoscopic coronary artery bypass; TECAB) [9, 10]. The field of minimally invasive coronary surgery quickly began to develop and a wide diversity of terms was introduced to distinguish each unique method. Often different terms have been used to describe a specific part of the procedure, focusing on the site of the incision, the technique for mammary artery harvest and the use of cardiopulmonary bypass (Fig. 1). The goal of this review was to provide a comprehensive overview of the different terms used for minimally invasive coronary bypass grafting throughout the years.

## METHODS

### Ethical statement

This review article does not require ethical approval or informed consent because it synthesizes publicly available literature and data from previously published studies.

### Literature search and eligibility criteria

**Figure 2:**
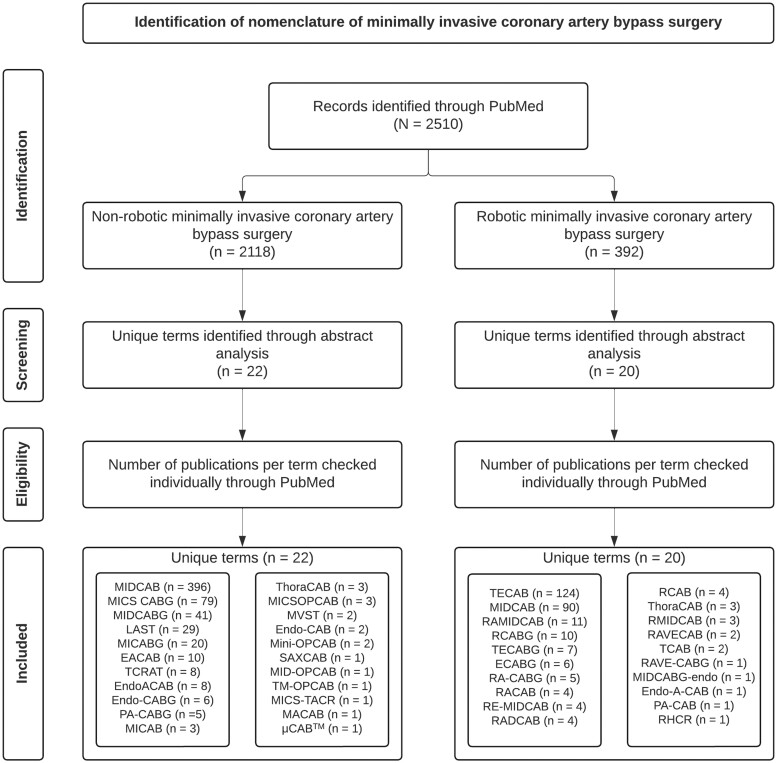
The search strategy illustrated in the Preferred Reporting Items for Systematic Reviews and Meta-Analyses, or PRISMA, diagram. A total of 40 unique terms were identified and included. EACAB: endoscopic atraumatic coronary artery bypass; ECABG: endoscopic coronary artery bypass grafting; EndoACAB: endoscopic atraumatic coronary artery bypass grafting; Endo-A-CAB: minimally invasive coronary artery bypass; Endo-CAB: endoscopic coronary artery bypass grafting; Endo-CABG: endoscopic coronary artery bypass grafting; LAST: left anterior small thoracotomy; MACAB: minimally invasive off-pump anaortic coronary artery bypass; MICS CABG: minimally invasive cardiac surgery; MICSOPCAB: minimally invasive cardiac surgery off-pump coronary artery bypass; MICS-TACR: minimally invasive coronary surgery–total arterial coronary revascularization; MICAB: minimally invasive coronary artery bypass; MICABG: minimally invasive coronary artery bypass grafting; MIDCAB: minimally invasive direct coronary artery bypass grafting; MIDCABG: minimally invasive direct coronary artery bypass grafting; MIDCABG-endo: endoscopic minimally invasive direct coronary artery bypass grafting; MID-OPCAB: minimally invasive direct off-pump coronary artery bypass grafting; Mini-OPCAB: mini off-pump coronary artery bypass; MVST: no-touch aorta multivessel small thoracotomy coronary artery bypass grafting; PA-CAB: port access coronary artery bypass; PA-CABG: port-access coronary artery bypass grafting; RADCAB: robotic-assisted minimally invasive direct coronary artery bypass; RA-CABG: robotic-assisted coronary artery bypass grafting; RACAB: robotic-assisted coronary artery bypass; RAMIDCAB: robotic-assisted minimally invasive direct coronary artery bypass; RAVECAB: robot-assisted, video-enhanced coronary artery bypass; RAVECABG: robot-assisted, video-enhanced coronary artery bypass grafting; RCAB: robotic coronary artery bypass; RCABG: robotic coronary artery bypass grafting; RE-MIDCAB: robotically enhanced minimally invasive direct coronary artery bypass; RHCR: robotic hybrid coronary revascularization; RMIDCAB: robotic-assisted minimally invasive direct coronary artery bypass; SAX-CAB: subclavian/axillary artery to coronary artery bypass; TCAB: transabdominal endoscopic computer-enhanced coronary artery bypass; TCRAT: total coronary revascularization through left anterior thoracotomy; TECAB: closed-chest totally endoscopic coronary artery bypass; TECABG: totally endoscopic coronary artery bypass grafting; ThoraCAB: robot-assisted minimally invasive direct coronary artery bypass grafting; TM-OPCAB: lower distal mini-sternotomy; µCAB: customized technology for subxiphoid internal mammary artery harvest.

A literature search was performed by both authors in August 2024 using the PubMed electronic database. To extract the best search results “minimally invasive” and “coronary artery bypass grafting” were used as either keywords or MeSH terms. The term *robotic* was specifically included for the second search, focusing on robotic-assisted procedures. The search strategy is shown in the Preferred Reporting Items for Systematic Reviews and Meta-Analyses, or PRISMA, diagram in Fig. 2. Eligible articles for this review were articles using an abbreviation to describe minimally invasive coronary artery bypass grafting. An additional search was performed using this abbreviation. If the abbreviation alone did not yield specific enough results, an additional MeSH term *coronary artery bypass grafting* was added. The search results were again screened to filter out the articles mentioning the specific abbreviation concerning minimally invasive coronary surgery.

## RESULTS

**Figure 3:**
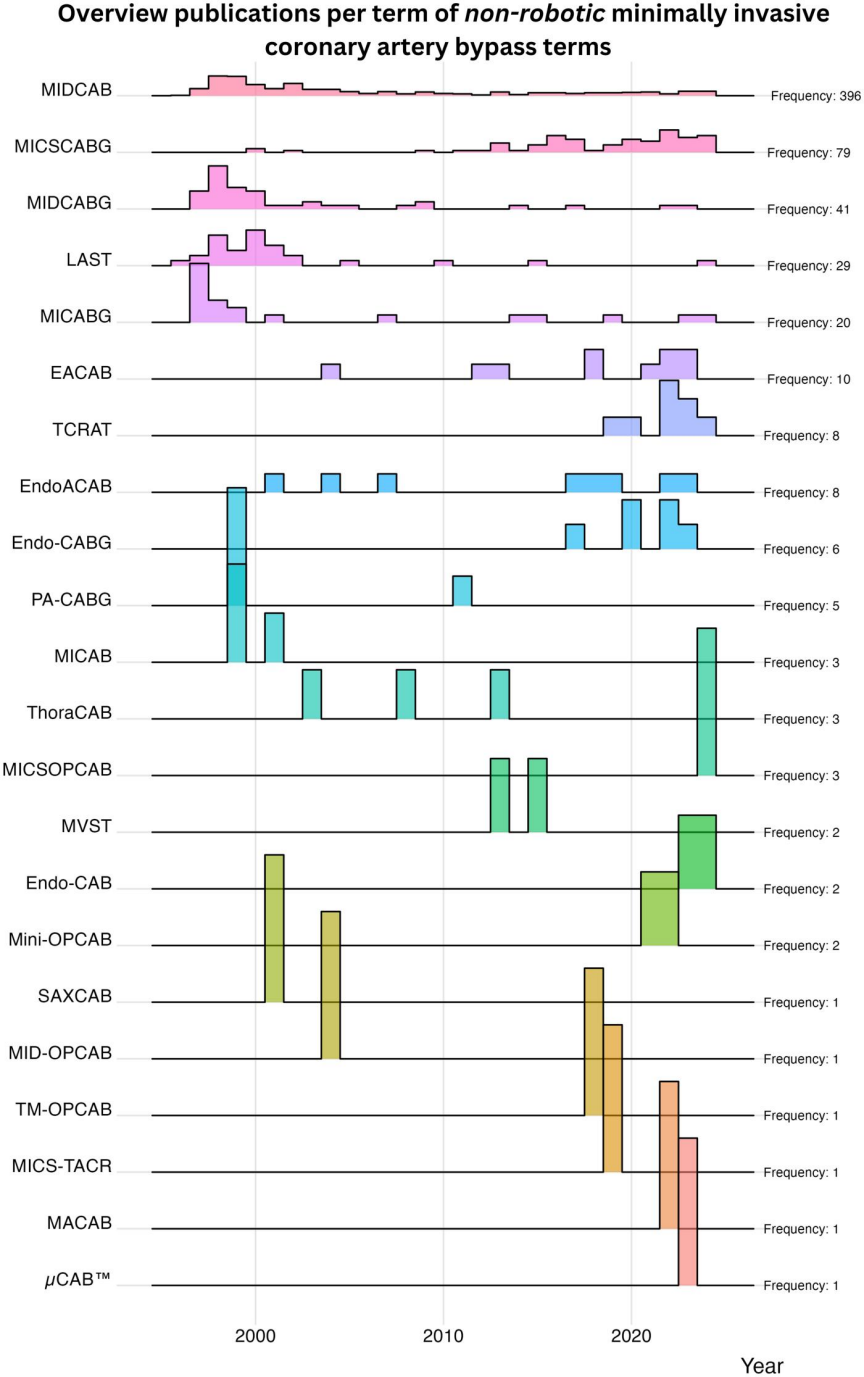
Overview of publications over the years per term of non-robotic minimally invasive coronary artery bypass approaches. EACAB: endoscopic atraumatic coronary artery bypass; EndoACAB: endoscopic atraumatic coronary artery bypass grafting; Endo-CAB: endoscopic coronary artery bypass grafting; Endo-CABG: endoscopic coronary artery bypass grafting; LAST: left anterior small thoracotomy; MACAB: minimally invasive off-pump anaortic coronary artery bypass; MICS CABG: minimally invasive cardiac surgery; MICSOPCAB: minimally invasive cardiac surgery off-pump coronary artery bypass; MICS-TACR: minimally invasive coronary surgery-total arterial coronary revascularization; MICAB: minimally invasive coronary artery bypass; MICABG: minimally invasive coronary artery bypass grafting; MIDCAB: minimally invasive direct coronary artery bypass grafting; MIDCABG: minimally invasive direct coronary artery bypass grafting; MID-OPCAB: minimally invasive direct off-pump coronary artery bypass grafting; Mini-OPCAB: mini off-pump coronary artery bypass; MVST: no-touch aorta multivessel small thoracotomy coronary artery bypass grafting; PA-CABG: port-access coronary artery bypass grafting; SAX-CAB: subclavian/axillary artery to coronary artery bypass; TCRAT: total coronary revascularization through left anterior thoracotomy; ThoraCAB: robot-assisted minimally invasive direct coronary artery bypass grafting; TM-OPCAB: lower distal mini-sternotomy; µCAB: customized technology for subxiphoid internal mammary artery harvest.

**Figure 4:**
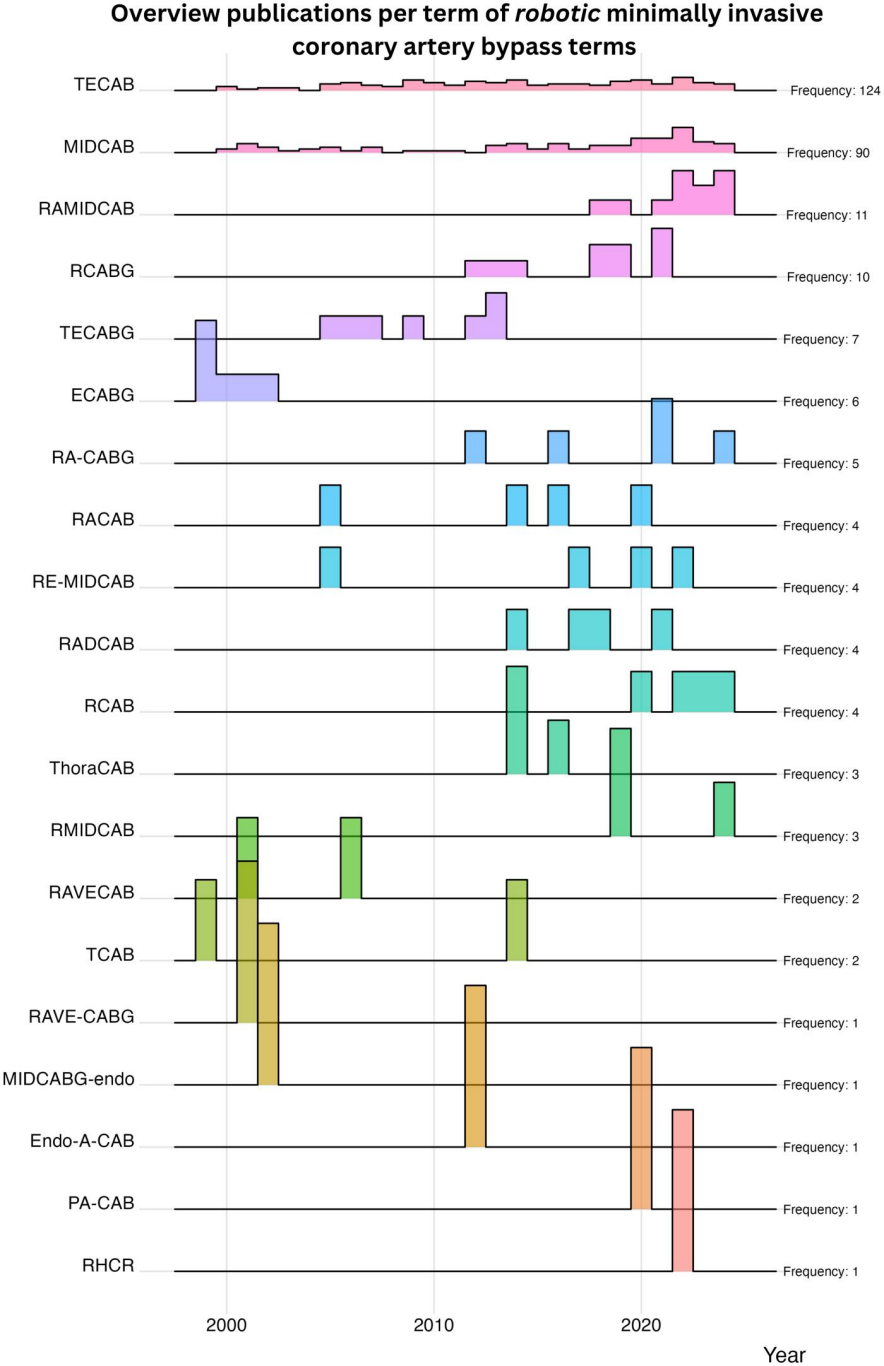
Overview of publications over the years per term of robotic minimally invasive coronary artery bypass approaches. ECABG: endoscopic coronary artery bypass grafting; Endo-A-CAB: minimally invasive coronary artery bypass; MIDCAB: minimally invasive direct coronary artery bypass grafting; MIDCABG-endo: endoscopic minimally invasive direct coronary artery bypass grafting; PA-CAB: port access coronary artery bypass; RADCAB: robotic-assisted minimally invasive direct coronary artery bypass; RA-CABG: robotic-assisted coronary artery bypass grafting; RACAB: robotic-assisted coronary artery bypass; RAMIDCAB: robotic-assisted minimally invasive direct coronary artery bypass; RAVECAB: robot-assisted, video-enhanced coronary artery bypass; RAVECABG: robot-assisted, video-enhanced coronary artery bypass grafting; RCAB: robotic coronary artery bypass; RCABG: robotic coronary artery bypass grafting; RE-MIDCAB: robotically enhanced minimally invasive direct coronary artery bypass; RHCR: robotic hybrid coronary revascularization; RMIDCAB: robotic-assisted minimally invasive direct coronary artery bypass; TCAB: transabdominal endoscopic computer-enhanced coronary artery bypass; TECAB: closed-chest totally endoscopic coronary artery bypass; TECABG: totally endoscopic coronary artery bypass grafting; ThoraCAB: robot-assisted minimally invasive direct coronary artery bypass grafting.

A total of 2118 publications were included describing 22 different techniques for minimally invasive coronary procedures without robotic assistance. The first publication was from 1996, and the last was from 2024. For robotic-assisted coronary procedures, 392 publications were included describing 20 different techniques. After the unique terms were identified, the number of publications per term was counted (Figs. 2–4). Minimally invasive direct coronary artery bypass grafting (MIDCAB) was the most studied (486 publications, non-robotic and robotic), followed by totally endoscopic coronary artery bypass grafting (TECAB) (124 publications). There were a total of 11 unique terms with a single publication each. Tables 1 and 2 provide an overview of the technical details of non-robotic terms. Figures 3 and 4 show ridgeline plots displaying the densities of the publications for each term per year.

**Table 1:** Overview of the technical specifications per technique—*non-robotic*.

Term	First article	Author	Single vessel	Multi vessel	Endoscopic IMA	Direct vision IMA	On-pump	Off-pump	Type of incision	Article count
EACAB	2004	Cisowski *et al.* [11]	Yes	No	Yes	No	No	Yes	Mini-thoracotomy	10
EndoACAB	2001	Vassiliades *et al.* [12]	Yes	Yes	Yes	No	No	Yes	Mini-thoracotomy	8
Endo-CAB	2023	Akca *et al.* [13]	Yes	Yes	Yes	No	No	Yes	Mini-thoracotomy	2
Endo-CABG	2017	Nijs *et al.* [14]	Yes	Yes	Yes	No	Yes	No	Mini-thoracotomy	6
LAST	1996	Calafiore *et al.* [7]	Yes	Yes	No	Yes	Yes	Yes	Mini-thoracotomy	29
MACAB	2022	Mavioglu *et al.* [15]	No	Yes	No	Yes	No	Yes	Mini-thoracotomy	1
MICS CABG	2000	Kitamura *et al.* [16]	Yes	Yes	No	Yes	Yes	Yes	Mini-thoracotomy	79
MICS-TACR	2019	Guo *et al.* [17]	No	Yes	No	Yes	No	Yes	Mini-thoracotomy	1
MICSOPCAB	2024	Ushioda *et al.* [18]	Yes	Yes	No	Yes	No	Yes	Mini-thoracotomy	3
MICAB	1998	Burgwardt *et al.* [19]	Yes	Yes	Yes	No	No	Yes	Mini-thoracotomy	3
MICABG	1997	Jatene *et al.* [20]	Yes	Yes	Yes	Yes	No	Yes	Mini-thoracotomy	20
MIDCAB	1996	Greenspun *et al.* [8]	Yes	Yes	No	Yes	Yes	Yes	Mini-thoracotomy	396
MIDCABG	1997	Arom *et al.* [21]	Yes	Yes	No	Yes	Yes	Yes	Mini-thoracotomy	41
MID-OPCAB	2004	Toumpoulis *et al.* [22]	Yes	Yes	No	Yes	No	Yes	Mini-thoracotomy + lower hemisternotomy	1
Mini-OPCAB	2021	Benetti *et al.* [23]	Yes	Yes	No	Yes	No	Yes	Mini-thoracotomy	2
MVST	2012	Une *et al.* [24]	No	Yes	Yes	No	Yes	Yes	Mini-thoracotomy	2
PA-CABG	1999	Galloway *et al.* [25]	Yes	Yes	Yes	No	Yes	No	Mini-thoracotomy	5
SAX-CAB	2001	Coulson *et al.* [26]	Yes	Yes	No	Yes	Yes	Yes	Mini-thoracotomy + clavicular	1
TCRAT	2019	Babliak *et al.* [27]	Yes	Yes	No	Yes	Yes	No	Mini-thoracotomy	8
ThoraCAB	2003	Srivastava *et al.* [28]	Yes	Yes	No	Yes	Yes	Yes	Mini-thoracotomy	3
TM-OPCAB	2018	Su *et al.* [29]	No	Yes	No	Yes	No	Yes	Mini-thoracotomy	1
µCAB	2023	Živković *et al.* [30]	Yes	Yes	Yes	No	No	Yes	Mini-thoracotomy + subxyphoid incision	1

EACAB: endoscopic atraumatic coronary artery bypass; EndoACAB: endoscopic atraumatic coronary artery bypass grafting; Endo-CAB: endoscopic coronary artery bypass grafting; Endo-CABG: endoscopic coronary artery bypass grafting; LAST: left anterior small thoracotomy; MACAB: minimally invasive off-pump anaortic coronary artery bypass; MICS CABG: minimally invasive cardiac bypass surgery; MICSOPCAB: minimally invasive cardiac surgery off-pump coronary artery bypass; MICS-TACR: minimally invasive coronary surgery–total arterial coronary revascularization; MICAB: minimally invasive coronary artery bypass; MICABG: minimally invasive coronary artery bypass grafting; MIDCAB: minimally invasive direct coronary artery bypass grafting; MIDCABG: minimally invasive direct coronary artery bypass grafting; MID-OPCAB: minimally invasive direct off-pump coronary artery bypass grafting; Mini-OPCAB: mini off-pump coronary artery bypass; MVST: no-touch aorta multivessel small thoracotomy coronary artery bypass grafting; PA-CABG: port-access coronary artery bypass grafting; SAX-CAB: subclavian/axillary artery to coronary artery bypass; TCRAT: total coronary revascularization through left anterior thoracotomy; ThoraCAB: robot-assisted minimally invasive direct coronary artery bypass grafting; TM-OPCAB: lower distal mini-sternotomy; µCAB: customized technology for subxiphoid internal mammary artery harvest.

**Table 2:** Overview of the technical specifications per technique—*robotic*.

Term	First article	Author	Single vessel	Multi vessel	Robotic IMA	Closed chest	On-pump	Off-pump	Type of incision	Article count
ECABG	1999	Ducko *et al.* [10]	Yes	No	Yes	Yes	Yes	No	Totally endoscopic	6
Endo-A-CAB	2012	Wu *et al.* [31]	Yes	No	Yes	No	No	Yes	Mini-thoracotomy	1
MIDCAB	2000	Nataf *et al.* [32]	Yes	Yes	Yes	No	Yes	Yes	Mini-thoracotomy	90
MIDCABG-endo	2002	Bucerius *et al.* [33]	Yes	No	Yes	No	No	Yes	Mini-thoracotomy	1
PA-CAB	2020	Hammal *et al.* [34]	Yes	No	Yes	Yes	Yes	No	Totally endoscopic	1
RA-CABG	2012	Cho *et al.* [35]	Yes	No	Yes	No	Yes	Yes	Mini-thoracotomy	5
RACAB	2005	Derose *et al.* [36]	Yes	No	Yes	No	No	Yes	Mini-thoracotomy	4
RADCAB	2014	Sabashnikov *et al.* [37]	Yes	No	Yes	No	Yes	No	Mini-thoracotomy	4
RAMIDCAB	2018	Pasrija *et al.* [38]	Yes	No	Yes	No	No	Yes	Mini-thoracotomy	11
RAVECAB	2001	Boyd *et al.* [39]	Yes	No	Yes	No	No	Yes	Mini-thoracotomy	2
RAVE-CABG	2001	Tang *et al.* [40]	Yes	No	Yes	No	No	Yes	Mini-thoracotomy	1
RCAB	2020	Hammal *et al.* [34]	Yes	No	Yes	No	Yes	No	Mini-thoracotomy	4
RCABG	2012	Kiani *et al.* [41]	Yes	Yes	Yes	No	Yes	Yes	Mini-thoracotomy	10
RE-MIDCAB	2005	Davidavicius *et al.* [42]	Yes	No	Yes	No	No	Yes	Mini-thoracotomy	4
RHCR	2022	Aluthman *et al.* [43]	Yes	No	Yes	No	No	Yes	Mini-thoracotomy	1
RMIDCAB	2019	Nakamura *et al.* [44]	Yes	No	Yes	No	No	Yes	Mini-thoracotomy	3
TCAB	1999	Falk *et al.* [45]	Yes	No	Yes	Yes	No	Yes	Totally endoscopic	2
TECAB	2000	Falk *et al.* [46]	Yes	Yes	Yes	Yes	Yes	No	Totally endoscopic	124
TECABG	2005	Cuvillon *et al.* [47]	Yes	Yes	Yes	Yes	Yes	Yes	Totally endoscopic	7
ThoraCAB	2014	Ishikawa *et al.* [48]	Yes	No	Yes	No	Yes	Yes	Mini-thoracotomy	3

ECABG: endoscopic coronary artery bypass grafting; Endo-A-CAB: minimally invasive coronary artery bypass; MIDCAB: minimally invasive direct coronary artery bypass grafting; MIDCABG-endo: endoscopic minimally invasive direct coronary artery bypass grafting; PA-CAB: port access coronary artery bypass; RADCAB: robotic-assisted minimally invasive direct coronary artery bypass; RA-CABG: robotic-assisted coronary artery bypass grafting; RACAB: robotic-assisted coronary artery bypass; RAMIDCAB: robotic-assisted minimally invasive direct coronary artery bypass; RAVECAB: robot-assisted, video-enhanced coronary artery bypass; RAVECABG: robot-assisted, video-enhanced coronary artery bypass grafting; RCAB: robotic coronary artery bypass; RCABG: robotic coronary artery bypass grafting; RE-MIDCAB: robotically enhanced minimally invasive direct coronary artery bypass; RHCR: robotic hybrid coronary revascularization; RMIDCAB: robotic-assisted minimally invasive direct coronary artery bypass; TCAB: transabdominal endoscopic computer-enhanced coronary artery bypass; TECAB: closed-chest totally endoscopic coronary artery bypass; TECABG: totally endoscopic coronary artery bypass grafting; ThoraCAB: robot-assisted minimally invasive direct coronary artery bypass grafting.

**Figure 5:**
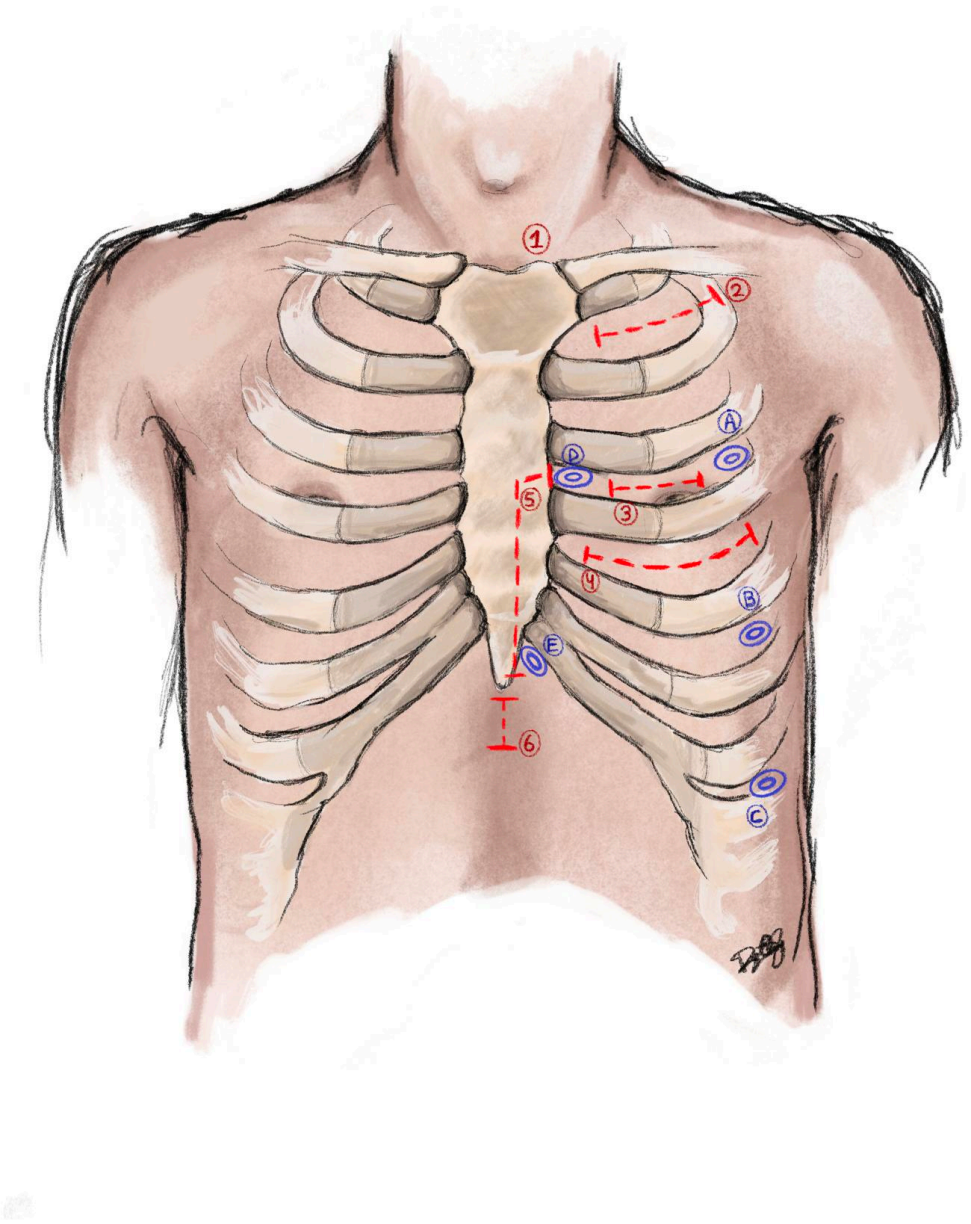
Visualization of the different incisions and port-access points used during minimally invasive coronary surgery. Incisions: (**1**) Subclavian/axillary artery to coronary artery bypass clavicular incision; (**2**) third intercostal mini-thoracotomy incision; (**3**) left anterior small thoracotomy or minimally invasive coronary artery bypass; (**4**) partial inferior hemi-sternotomy for lower distal mini-sternotomy; (**5**) internal mammary artery harvest incision for customized technology for subxiphoid internal mammary artery harvest. (**A–C**) Non-robotic and robotic endoscopic internal mammary artery harvest port placement locations; (**D, E**) robotic closed-chest revascularization port placement locations.

The results can be roughly classified into 4 groups. Three of the groups are based on the technique used to harvest the left internal mammary artery (LIMA), and the fourth is closed-chest total endoscopic revascularization: (i) In the first category, both the LIMA harvest and the anastomosis are performed directly through the thoracotomy (*n* = 586). (ii) The second category includes endoscopic-assisted LIMA harvest and a mini-thoracotomy for the anastomoses (*n* = 37). (iii) The third group comprises robotic LIMA harvest and the anastomoses through the mini-thoracotomy (*n* = 144). (iv) The fourth group includes the totally endoscopic robotic closed-chest group (*n* = 140). Figure 5 shows an overview of the different incisions and port-access points used during minimally invasive coronary surgery.

## DESCRIPTION OF MINIMALLY INVASIVE TECHNIQUES

### First publications

The LAST approach is the starting point of minimally invasive coronary surgery. The approach itself was pioneered by Benetti in the early 1990s [6]. The term *LAST* was first mentioned in a publication by Calafiore and Angelini in January 1996 in the *Lancet* [7]. A LIMA-to-LAD anastomosis through a fourth intercostal incision located left of the sternum is described, and a transthoracic pulsed wave Doppler was used to assess the patency of the graft during surgery [7]. The LAST method soon became widely accepted in clinical practice. The internal mammary arteries (IMAs) could be harvested under direct vision or endoscopically, and the anastomoses were made through the (mini)-thoracotomy. In later publications, LAST mainly refers to the mini-thoracotomy itself and not to the specifics of the grafting technique utilized [49]. The term MIDCAB (minimally invasive direct coronary artery bypass grafting) was introduced shortly after the first publication of LAST in 1996 by Greenspu *et al.* [8] After the first mention of MIDCAB, the term became widely adopted in clinical practice to refer to any minimally invasive coronary revascularization procedure. We categorized the different nomenclatures based on the mammary artery harvesting technique (direct vision, endoscopic-assisted or robotic-assisted) or fully closed-chest procedures.

### Direct vision

**Figure 6:**
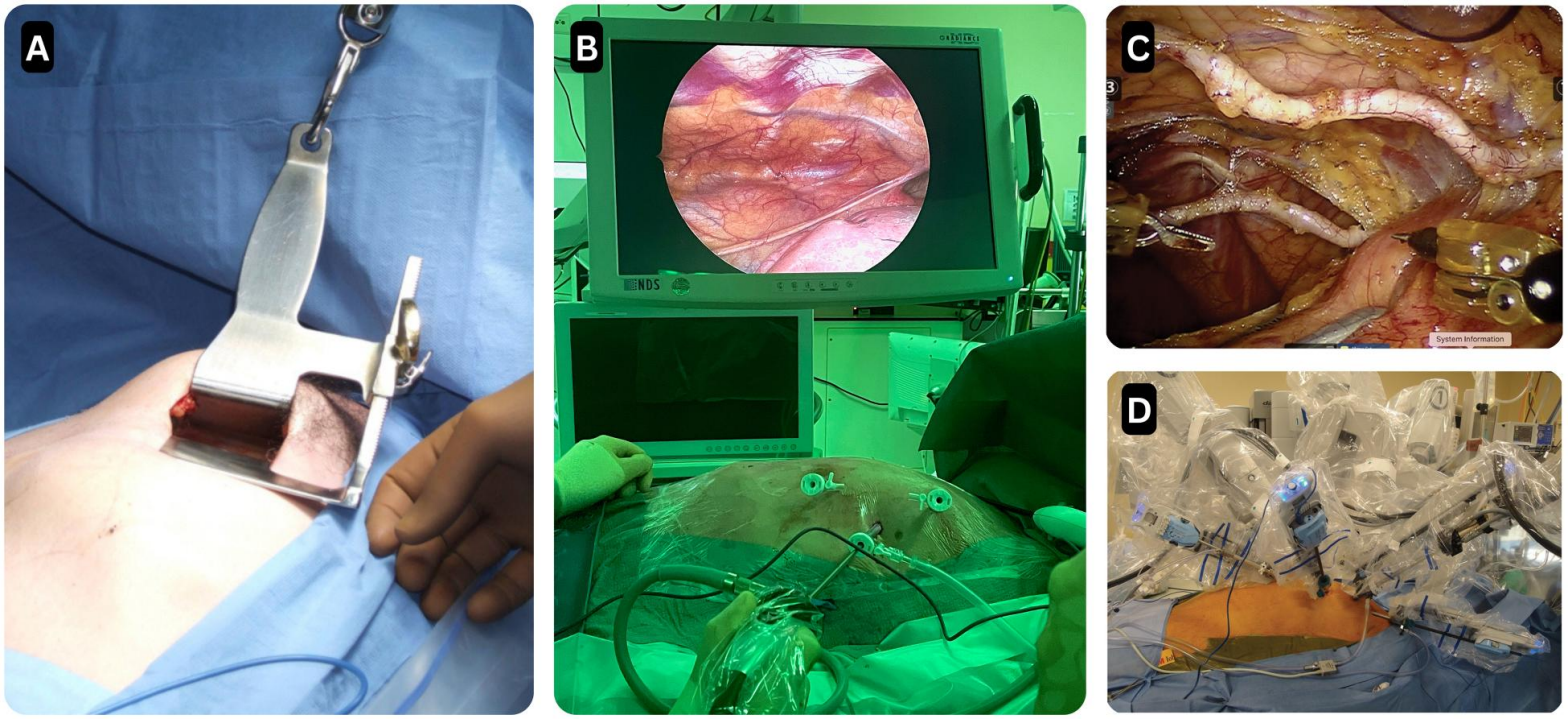
Pictures illustrating the different approaches to minimally invasive internal mammary artery harvest. (**A**) Illustrates direct vision harvest as described by Ruel [50]; (**B**) illustrates the non-robotic endoscopic assisted internal mammary artery harvest as performed at our institute; (**C**) and (**D**) illustrate the robotic-assisted internal mammary artery harvest set-up and the internal mammary artery harvest as described by Torregrossa and Balkhy [51, 52].

The location of the mini-thoracotomy varies per patient-specific anatomy or the surgeon’s preference. Generally, the incision (4–6 cm) is made between the fourth or fifth intercostal space on the mid-axillary line (Fig. 5). A special retractor is used to harvest the internal mammary artery (IMA) under direct vision (Fig. 6A). Živković and colleagues described a specially designed retractor for subxiphoid access for IMA harvest, naming the approach µCAB [30]. Another variation of the placement of the mini-thoracotomy is described by Su and colleagues to ease the access to the more distally located coronary targets with the lower distal mini-sternotomy (TM-OPCAB) [29].

The subclavian/axillary artery-to-coronary artery bypass (SAXCAB) was initially developed as a rescue procedure in situations where an aortic approach was not possible and the IMAs were unusable due to previous surgical damage or underdevelopment. With this approach, the subclavian or axillary artery is used as an inflow source avoiding aortic manipulation and the risk of postoperative stroke. The subclavian/axillary artery for a proximal anastomosis can be accessed both through a median sternotomy or through a mini-thoracotomy with an additional intra-clavicular incision. A vein-to-coronary artery configuration can be accomplished through the incision. In the review of Coulson and colleagues, they suggest that placing the venous graft in parallel to the IMA provides protective haemodynamic qualities [26].

The anastomoses can be made using off-pump techniques or on an arrested heart. If the grafting occurs with a beating heart, cardiac stabilizing devices are used [15]. Anaortic or no-touch aorta off-pump coronary bypass grafting is associated with a reduction in neurological events and is described by Mavioglu *et al.* as minimally invasive off-pump anaortic coronary artery bypass (MACAB) [15,53]. If the lateral and inferior territories need to be grafted using off-pump techniques, cardiac stabilizers are used as described by Kikuchi *et al.* during minimally invasive coronary artery bypass grafting (MICS CABG) [54]. If the grafting process is performed on an arrested heart through the mini-thoracotomy, an aortic cross-clamp is placed and cardioplegia is administered. This situation is described by Yilmaz *et al.* as endo-CABG (endoscopic coronary artery bypass grafting) and by Babliak *et al.* as total coronary revascularization through a left anterior thoracotomy (TCRAT) [27, 55].

### Endoscopic-assisted procedures

Endoscopic-assisted IMA harvest and grafting through a mini-thoracotomy have been described for both single-vessel and multivessel revascularization (Fig. 6A and 6B). The use of both venous and arterial conduits has been documented. For arterial conduits, endoscopically harvested IMA, gastroepiploic artery and radial arteries have been described [56, 57].

Vassiliades was the first to describe an endoscopic-assisted IMA harvest in conjunction with a mini-thoracotomy in 2001, named EndoACAB (endoscopic atraumatic coronary artery bypass). He describes an endoscopic IMA takedown approach using a voice-activated endoscope. The endoscopic ports avoid excessive rib spreading compared to direct vision harvest retractors [12]. Endoscopic ports are positioned around the third, fifth and seventh intercostal spaces, between the mid and anterior axillary lines. After initiating unilateral ventilation, 1 or both IMAs can be harvested under CO_2_ insufflation. Once the IMAs are harvested, the coronary target is marked and the mini-thoracotomy is created [58]. A needle can be used to mark the location of the mini-thoracotomy as described during minimally invasive coronary artery bypass (MICAB) by Ohtsuka *et al.* [59].

After an endoscopic IMA takedown, the grafts can be made on a beating or an arrested heart through a mini-thoracotomy. When the anastomoses are made with the beating heart using off-pump techniques, cardiac stabilizers are used. If the anastomoses are made with an arrested heart, peripheral cardiopulmonary bypass is initiated with a beating heart or an arrested heart [55].

### Robotically assisted procedures

Port-access IMA harvest made it possible to adapt IMA harvest instruments for robotic surgical systems (Fig. 6C and 6D). Robotic-assisted minimally invasive coronary artery bypass grafting was performed first in the late 1990s by Mohr *et al.* and Reichenspurner *et al.* [9, 10, 60] The surgical robot is used to harvest the IMA through endoscopic ports, and the IMA can be obtained with minimal chest wall trauma. After the IMA is obtained, the anastomoses can be made through a mini-thoracotomy [42, 43]. Terms such as MIDCAB (minimally direct coronary artery bypass grafting) and robotic-assisted minimally invasive direct coronary artery bypass (RAMIDCAB) have been used to refer to robotically assisted procedures.

The patient is placed in a supine position with a slight elevation of the left hemithorax for the LIMA harvest. A total of 3 ports are placed at the third, fifth and seventh intercostal spaces to facilitate the camera, CO_2_ insufflation and robotic instruments. CO_2_ insufflation is similar to that used for non-robotic IMA harvest. A 30-degree camera is used to provide visualization during harvest. Fine tissue forceps and cautery tools are used to dissect the LIMA [61]. After the robotic harvest of the IMA, the location of the mini-thoracotomy is marked and the robot is undocked. The mini-thoracotomy is made, and the anastomoses can be made on a beating or an arrested heart as described previously. Graft conduits obtained elsewhere can be utilized to treat multivessel disease. Ishikawa *et al.* [48] describe robot-assisted minimally invasive direct coronary artery bypass grafting (ThoraCAB) multivessel procedures.

### Closed-chest procedures

Complete closed-chest robotic-assisted revascularization is the least invasive option of all the minimally invasive surgical techniques currently available. Animal feasibility studies were performed to see if both the IMA harvest and the anastomosis could be performed completely endoscopically (ECABG, endoscopic coronary artery bypass grafting) [10]. The first closed-chest, robotic-assisted coronary bypass procedure was performed by Mohr *et al.* using the da Vinci system in May 1998 [9]. Both the IMA harvest and the anastomoses are performed completely endoscopically during TECAB (closed-chest totally endoscopic coronary artery bypass). Both on-pump and off-pump techniques can be used to treat single and multivessel disease. For TECAB procedures, additional parasternal and subcostal assistance ports are placed to facilitate the robotic anastomosis. The inaccessibility of robotic-compatible cardiac stabilizers endangers the future of off-pump totally endoscopic robotic procedures [62].

### Conflicting terminology

Finding a term that describes all the different technical details of minimally invasive coronary surgery is impossible. Making a distinction between the placement of the incision, the materials used and the specific technique is difficult to capture in 1 abbreviation. If a more general term were used instead, a significant loss in detail could occur that might lead to confusion. However, the introduction of 40 unique terms referring to minimally invasive coronary surgery might be extremely confusing as well, also for the referring cardiologist.

The term *minimally invasive direct coronary artery bypass grafting* (MIDCAB) in the literature can refer to both robotic and non-robotic-assisted revascularization. In the case of robotic assistance, the IMA is harvested robotically, and the grafting occurs through a mini-thoracotomy [8, 63]. Another term that has been used for both robotic and non-robotic approaches is thoracoscopic coronary artery bypass (ThoraCAB). Although ThoraCAB was first used to refer to non-robotic approaches, more recently it has been used exclusively for robotic-assisted revascularization [28, 48]. This term is used much less frequently and illustrates the possible confusion when the exact technique being referred to is not clear.

Looking at the prefixes, terminology for robotic techniques tends to be more descriptive than for non-robotic procedures. Prefixes starting with an “R” give a clear indication that it is a robotic technique. Likewise, the prefix “TE” used in “TECAB” and “TECABG” encompasses the robotic-assisted IMA harvest and total endoscopic bypass grafting. At present, total endoscopic revascularization can only be accomplished using robotic assistance, so the reference is clear. In contrast, the prefixes “MID”, “MICS” and “MI” merely refer to the minimally invasive aspect and do not clarify the IMA harvest technique or site of incision. For an endoscopic IMA harvest, the prefix “Endo” or “PA” is frequently used, referring to endoscopic and port-assisted, respectively.

### Minimally invasive coronary surgery classification

No subspecialty in cardiac surgery has as many abbreviations as does coronary surgery. Based on the available techniques for the site of incision (*n* = 4), techniques for mammary artery harvest (*n* = 3) and use of cardiopulmonary bypass (*n* = 2), a total of 24 combinations are possible (Fig. 1). Nevertheless, 40 unique terms are described in the literature for these procedures. Often these terms are used to distinguish the described technique from previously published procedures, often with many similarities. For anyone involved in the field of minimally invasive coronary surgery, it is important to know these described techniques and terms.

## CONCLUSIONS

A wide variety of terms are used within the field of minimally invasive coronary surgery. A total of 40 different terms are published, each describing certain specifics of the procedure. The variety of the terminology underscores the wide diversity in approaches and techniques. Ultimately the 40 unique terms illustrate the progression and innovative nature of minimally invasive coronary surgery and are of importance for anyone involved in this field.

## Data Availability

Data will be made available by the corresponding author upon reasonable request.

## ABBREVIATIONS

CABG: Coronary artery bypass grafting
IMA: Internal mammary artery
LAST: Left anterior small thoracotomy
LIMA: Left internal mammary artery
MIDCAB: Minimally invasive direct coronary artery bypass
TECAB: Closed-chest totally endoscopic coronary artery bypass
TECABG: Totally endoscopic coronary artery bypass grafting
ThoraCAB: Robot-assisted minimally invasive direct coronary artery bypass grafting
TM-OPCAB: Lower distal mini-sternotomy
µCAB: Customized technology for subxiphoid internal mammary artery harvest
